# Antenatal counseling in maternal and newborn care: use of job aids to improve health worker performance and maternal understanding in Benin

**DOI:** 10.1186/1471-2393-10-75

**Published:** 2010-11-22

**Authors:** Larissa Jennings, André Sourou Yebadokpo, Jean Affo, Marthe Agbogbe

**Affiliations:** 1 USAID Health Care Improvement Project, University Research Co., LLC, Bethesda, Maryland, USA; 2 Department of Population, Family, and Reproductive Health, Johns Hopkins Bloomberg School of Public Health, Baltimore, Maryland, USA; 3 Integrated Family Health Project, University Research Co., LLC, Bohicon, Benin

## Abstract

**Background:**

Antenatal care provides an important opportunity to improve maternal understanding of care during and after pregnancy. Yet, studies suggest that communication is often insufficient. This research examined the effect of a job aids-focused intervention on quality of counseling and maternal understanding of care for mothers and newborns.

**Methods:**

Counseling job aids were developed to support provider communication to pregnant women. Fourteen health facilities were randomized to control or intervention, where providers were trained to use job aids and provided implementation support. Direct observation of antenatal counseling sessions and patient exit interviews were undertaken to assess quality of counseling and maternal knowledge. Providers were also interviewed regarding their perceptions of the tools. Data were collected before and after the job aids intervention and analyzed using a difference-in-differences analysis to quantify relative changes over time.

**Results:**

Mean percent of recommended messages provided to pregnant women significantly improved in the intervention arm as compared to the control arm in birth preparedness (difference-in-differences [Δ_I-C_] = +17.9, 95%CI: 6.7,29.1), danger sign recognition (Δ_I-C _= +26.0, 95%CI: 14.6,37.4), clean delivery (Δ_I-C _= +21.7, 95%CI: 10.9,32.6), and newborn care (Δ_I-C _= +26.2, 95%CI: 13.5,38.9). Significant gains were also observed in the mean percent of communication techniques applied (Δ_I-C _= +28.8, 95%CI: 22.5,35.2) and duration (minutes) of antenatal consultations (Δ_I-C _= +5.9, 95%CI: 3.0,8.8). No relative increase was found for messages relating to general prenatal care (Δ_I-C _= +8.2, 95%CI: -2.6,19.1). The proportion of pregnant women with correct knowledge also significantly improved for birth preparedness (Δ_I-C _= +23.6, 95%CI: 9.8,37.4), danger sign recognition (Δ_I-C _= +28.7, 95%CI: 14.2,43.2), and clean delivery (Δ_I-C _= +31.1, 95%CI: 19.4,42.9). There were no significant changes in maternal knowledge of general prenatal (Δ_I-C _= -6.4, 95%CI: -21.3,8.5) or newborn care (Δ_I-C _= +12.7, 95%CI: -6.1,31.5). Job aids were positively perceived by providers and pregnant women, although time constraints remained for health workers with other clinical responsibilities.

**Conclusions:**

This study demonstrates that a job aids-focused intervention can be integrated into routine antenatal care with positive outcomes on provider communication and maternal knowledge. Efforts are needed to address time constraints and other communication barriers, including introduction of on-going quality assessment for long-term sustainability.

## Background

Antenatal care provides an important opportunity to improve maternal understanding about pregnancy, childbirth, and care of the newborn. In addition to routine examination, screening, and treatment, the World Health Organization's focused antenatal care model recommends information and counseling be provided to all pregnant women in areas related to the health needs of the pregnant woman, birth and emergency preparedness, nutrition, preventative home practices, and support for care-seeking through danger sign recognition [1]. This includes advice that promotes the health of the mother and newborn during and following delivery. Relatively high coverage of antenatal care enables health care personnel to reinforce communication across visits [2].

Communication provided antenatally has been shown to be an effective strategy to improve maternal understanding and health practices [3,4]. Yet, in many developing countries, information is lacking on the intrinsic quality of communication, limiting one's ability to assess intervention effects [5]. Studies that have examined quality of antenatal counseling suggest that adequacy of information provided is low - with less information-sharing than guidelines recommend [6-8]. Available data suggests that patients often perceive counseling to be poor [9], and low maternal knowledge following counseling has been attributed to insufficient communication [10-12].

There is a growing need for implementation research in developing countries that examines content of antenatal communication and effective strategies to improve maternal and newborn care counseling. Such interventions hold promise for reducing maternal and neonatal mortality in resource-poor settings. Traditional efforts to improve provider communication have often relied on resource-intensive strategies such as off-site training or continuing medical education [13]. However, recent evidence shows that job aids can serve as an acceptable, low-cost alternative to improve health worker performance when combined with minimal training and supervision [14-18]. Job aids are support tools with written information that is often enhanced by images, making readily available information needed to comply with standards while minimizing provider dependence on memory [19,20]. In the context of counseling, pictorial job aids can strengthen communication by helping providers to remember key messages [21]. They may also function to facilitate communication processes by conveying ideas using imagery [22,23]. Job aids have been shown to be effective in areas such as malaria [14,15], infant feeding [16,24], and family planning [17,18]. Evidence is needed to examine use of job aids to improve antenatal communication in maternal and newborn care.

This study uses implementation research to assess quality of counseling provided to pregnant women and the impact of a job aids-focused intervention consisting of training, organizational change, and field support on provider communication and, in turn, maternal understanding. The study hypothesized that the quality of job aid-supported counseling would be better than that of counseling which was not supported, and that better counseling would yield higher levels of maternal understanding.

## Methods

### Study Design and Context

This study used a pre-post randomized group design. Data were collected from August to October 2008 in the Zou/Collines region of Benin. Antenatal care coverage is high in Benin and represents an ideal time to advise pregnant women. An estimated 88% of Beninese women receive at least one antenatal care visit, and 61% receive at least four visits [25]. Fourteen public health maternities were randomized to the intervention or control group. Women in the intervention group received counseling by nurse-midwives specifically trained in use of the job aids. Women in the control group received the usual care and advice. Data were obtained before and after introduction of the job aids in both control and intervention arms, yielding four cross-sectional groups.

### Sample selection

The target sample for each group was 154 pregnant women, sufficient to detect a mean difference of 15% percentage points in quality scores and proportional difference of 25% in maternal knowledge between groups at 80% power with an incompletion rate of 10% and a design effect of 2.0. Several public health centers were selected to achieve the target sample size and improve generalizability of results across sites, providers, and clients. All pregnant women presenting for antenatal consultation during the study period were eligible to participate. Using systematic sampling, eligible women were approached while waiting for consultation, given information regarding the purpose of the study, and invited to participate. As part of the information process, women were assured confidentiality and that opting out would not compromise the care they would receive. Participation from site managers and providers was obtained prior to the start of the study.

### Job Aids

**Figure 1:**
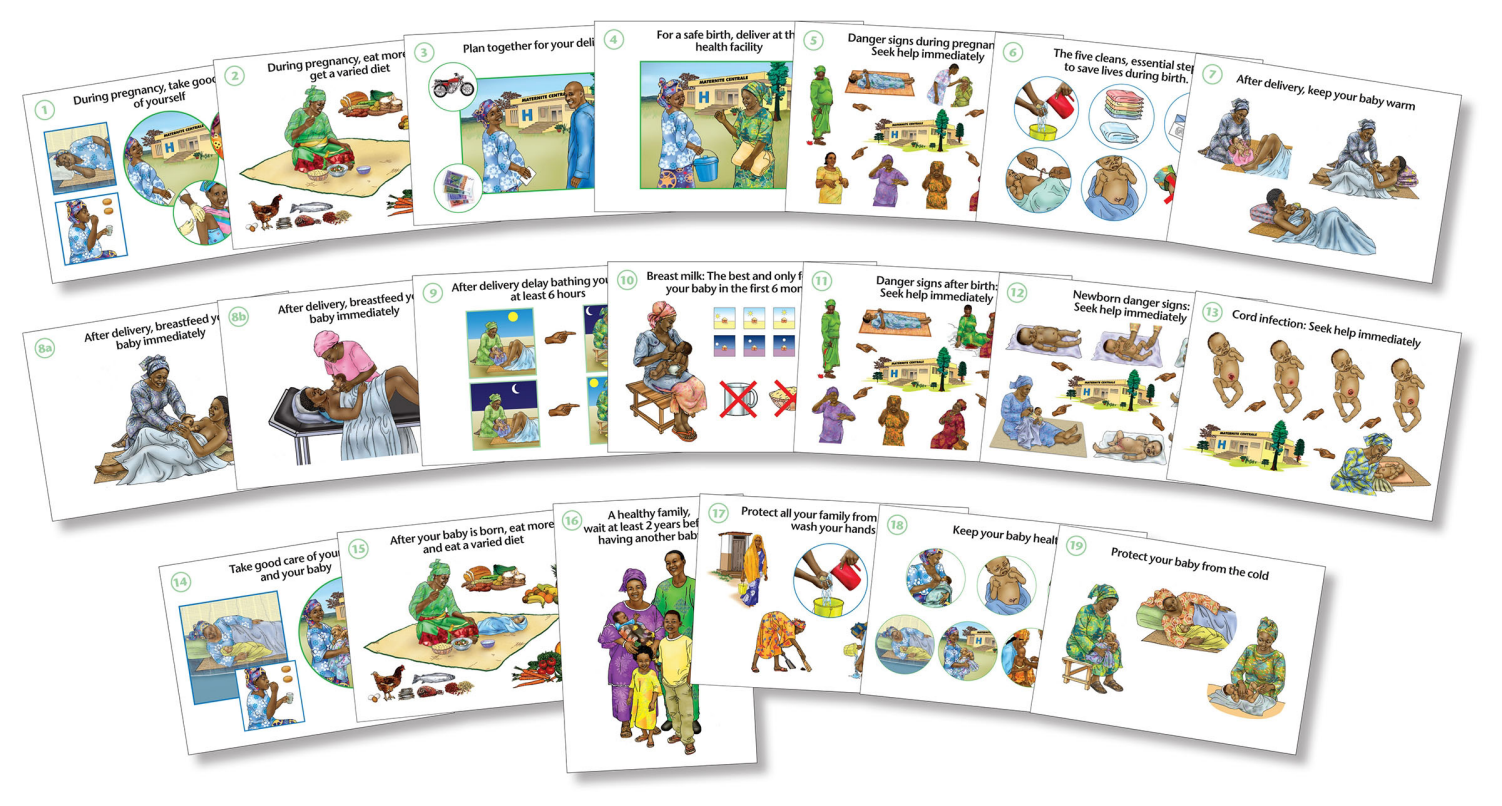
**Counseling job aids used for communication regarding pregnancy care, birth preparedness and maternal danger signs**. Actual size 8 × 11 (A1 sheet)

The job aids developed in this study were a set of pictorial counseling cards designed to support communication to women about care during and after pregnancy according to national guidelines [Figure 1]. Generic counseling cards and counseling materials for maternal and newborn care were used as a basis for their design by the USAID Quality Assurance Project (QAP) and the Integrated Family Health Project Health Project (PISAF), both managed by University Research Co., LLC (URC), in collaboration with the Benin Ministry of Health. Culturally appropriate images were designed based on community and subject expert feedback as well as a pilot field study. One side of the counseling card provided guidance to the provider on key messages to convey (the "job aid"). The other side of the card was a pictorial guide for the client that used illustrations to portray core messages (the "visual aid"). The overall objective of the counseling cards was to support information-sharing by the provider, enhance the transfer of knowledge to women, and depict recommended health behaviors.

Eleven antenatal cards were organized into three modules that prioritized messages according to a woman's stage of pregnancy (early, mid-, or late) to ensure that over the course of pregnancy, a woman would have multiple exposures to key messages. Module A consisted of five cards for women in early pregnancy and included messages on general pregnancy care, birth and emergency preparedness, maternal danger signs, and clean delivery. Module B consisted of four cards for women at mid-pregnancy with information on newborn care, such as immediate and exclusive breastfeeding, delayed bathing, and thermal protection. Module C consisted of two cards for women in late pregnancy that reinforced key aspects of maternal and newborn care. In Benin, counseling on immediate newborn care is typically performed postnatally. However, in this study, counseling on newborn care was additionally piloted antenatally in intervention sites for women in mid-to-late pregnancy. Verification of the content of Module A or Modules A and B was a prerequisite for introduction of the next module.

### Description of Intervention

Introduction of the job aids consisted of three intervention components: training, organizational changes, and field support. All health care personnel at the intervention sites were trained for three days in the content and use of the counseling cards, interpersonal communication, and quality improvement. The training included role-playing and didactic instruction with available written materials, such as a technical reference guide and other documents.

The role-playing used two methods: one-on-one role-playing with feedback from the trainer and role-playing in plenary with feedback from peers. Providers practiced conducting a counseling session with the counseling cards following a 10-step process: (1) present the topic to be discussed; (2) ask the woman what she already knows about the topic; (3) present the counseling card; (4) ask the woman what she sees on the card; (5) encourage the woman to describe what message she thinks the card conveys; (6) based on the woman's response, elaborate on messages using images provided on the card; (7) verify the woman's understanding by asking her to summarize key points; (8) encourage her to ask questions; (9) summarize any remaining messages; and (10) check the back of the card to be sure all messages have been discussed. Providers applied this process in using the counseling job aids for any one of the three modules.

At the end of the training session, participants in each site formed a team to identify organizational changes needed to implement job-aid supported counseling at their site, such as ensuring the availability of the cards, organizing them into modules, assigning roles, or in sites where group counseling was provided - stratifying sessions by pregnancy stage to reduce group sizes. Teams also convened an on-site organizational meeting to build consensus on the new communication strategy and to further identify best practices for organizing counseling at their site. Prior to data collection, all site-level teams received a supervisory visit from one of the counseling instructors and/or technical advisors. These visits included direct observation of antenatal consultations using the job aids with immediate feedback and technical support, as well as open discussion about difficulties encountered during implementation. The post-training field visits aimed to assist health workers in overcoming communication barriers and strengthen overall communication processes. None of the three intervention components were introduced to providers in the control arm. Usual care and advice in these sites did not consist of job aids or targeted communication based on stage of pregnancy.

### The Improvement Context

Several participating sites (five in the control arm and four in the intervention arm) were concurrently engaged in a quality improvement collaborative to strengthen clinical care in management of normal deliveries and obstetric complications. A collaborative is an improvement strategy that engages a group of health workers at various sites to jointly improve quality of care for a specific technical area through cross-sharing of organizational learning and best practices [26]. The improvement collaborative included prior technical training of skilled providers in essential obstetric and newborn care who met quarterly during "learning sessions" to discuss best practices. Although the collaborative improved many clinical aspects of care, no efforts had been made to address counseling to women during antenatal consultations. Thus, while study sites had varying quality improvement experiences (e.g., collaborative versus non-collaborative), they were comparable in their limited focus on strengthening counseling to women on maternal and newborn care. To improve generalizability, the job aids were introduced to both types of sites, and intervention components targeted competencies relating to communication regardless of participation in the clinical collaborative.

### Measurement

This study measured three outcomes: (1) quality of counseling provided to pregnant women; (2) provider perceptions regarding use of the job aids; and (3) women's knowledge of messages relating to maternal and newborn care.

To evaluate quality of counseling, the content of communication and counseling technique used by the provider were measured through direct observation using a pre-tested observation checklist developed by the study team for this purpose. The checklist contained items categorized in five topic areas: general prenatal care, birth preparedness, dangers signs, clean delivery, and newborn care. "General prenatal care" included four messages relating to prevention and treatment of malaria (use of an insecticide-treated mosquito net and antimalarials), iron/folate supplementation, having at least four antenatal visits, and diet and nutrition. "Birth preparedness" included seven messages on identifying a place of delivery, identifying a skilled birth attendant, organizing transportation, setting money aside, planning for emergencies, planning with a family member, and identifying a blood donor. "Danger signs" highlighted nine maternal symptoms that require care: vaginal bleeding, convulsions, fever, water loss, abdominal pains, severe headaches, blurred vision, swelling of limbs, and absence of or diminished fetal movement. "Clean delivery" consisted of two messages relating to providing a clean, plastic cloth for delivery and clean, dry towels for the mother and newborn. Six messages related to "newborn care": skin-to-skin contact, early breastfeeding, exclusive breastfeeding, delayed bathing, clean cord care, and thermal protection. For each item, the trained observer selected 'yes' (coded as 1) or 'no' (coded as 0) depending on whether the woman received information on that item during her antenatal visit. The mean percent of messages provided for each group was calculated based on the number of messages provided to a woman within each category out of the total number of recommended messages. All messages provided during group and/or individual sessions were included to represent overall communication during each woman's antenatal visit. Provider communication techniques were scored similarly across six communication techniques: presentation of the subject, posing of questions to determine current knowledge, use of visual aid(s), verification of understanding, motivation to adapt behaviors, and asking the woman if she had questions. Information on the context of the session was also collected, such as the mode of communication (e.g., group or individual), session duration, and primary language used.

Provider perceptions on use of the counseling cards were examined only in the intervention arm and after implementation of the job aids. Data were obtained using individual interviews. Each health worker was asked four questions (one closed and three open-ended): whether she thought the counseling job aids should be introduced in other sites to support communication; what she considered to be advantages and disadvantages in using the job aids; and what recommendations, if any, she had to improve their overall use and effectiveness. Responses were coded and analyzed by topic area. Information on provider demographic characteristics, such as age, education, qualification, years working at site, and years working in public health, was also obtained.

To assess maternal understanding, pregnant women were interviewed at the clinic prior to departure. Structured questionnaires were written in French and administered orally in the local language, Fon. Women were asked to indicate what they considered to be important components of care during and after pregnancy for the mother and newborn as well as what they considered to be danger signs that required urgent medical care. On average, exit interviews took 30 to 45 minutes to complete. Also obtained were the woman's age, months in pregnancy, education, number of previous antenatal visits, first-time visit status, and number of living children.

Pre-intervention data were obtained in August 2008, and post-intervention data were obtained in October 2008, three weeks following the introduction of the intervention. All data collection tools were reviewed and approved by local Beninese project staff to make sure they were clear, easy to follow, appropriate, and relevant for the local culture. The observation team received three days of training in counseling observation, interviewer techniques, and questionnaire completion, including a standardization session to minimize inter-observer variability. Pre-tested, standardized questionnaires with a detailed guide for data collectors were used with routine supervision of data collectors' instruments. Supervisors observed approximately 5% of counseling sessions and patient interviews for quality control and validation purposes.

### Statistical Analysis

Data were analyzed using STATA (Version 9.2, StataCorp, College Station, TX). Difference-in-differences analyses within multivariate linear regressions were used to account for differences in baseline quality of counseling between intervention and control groups based on an interaction term of study arm and time. In difference-in-differences analyses, the coefficient of the interaction term and its subsequent 95% confidence interval (CI) represent the difference in the change over time in the intervention group as compared to the change over time in the control group [27]. As a result, calculation of the intervention's effect size is adjusted for differences over time in the control arm [28].

Additionally, the study employed three-level hierarchal modeling techniques to account for the inherent correlation of observations. Pregnant women (level 1) were nested within providers (level 2) who were nested within sites (level 3). Hierarchal regression models are more suitable for clustered data than conventional regression analyses that underestimate standard errors by assuming that observations from the same sites or providers are independent [29]. Random effects were incorporated for provider- and site-level characteristics, and fixed effects were incorporated for patient characteristics that significantly varied between groups. Random effects hierarchal analyses aim to correct for correlation of observations and account for unmeasured differences in level-specific characteristics [30]. This technique was used since a means-as-outcomes regression model indicated that no site or provider characteristics had significant direct effects on quality of communication.

Maternal knowledge was measured based on the proportion of women with knowledge of at least three items within each topic area. Similar to analyses of quality of counseling, mixed hierarchal regression models using difference-in-difference analyses were used to adjust for nesting of observations and baseline level differences. This was coupled with multivariate analyses to control for confounding. Data were double entered using EpiData (Version 3.2) with automatic checks for quality control. In all analyses, the level of significance was considered at *p *≤ 0.05.

### Ethics Approval

This study received ethics approval by the Johns Hopkins Bloomberg School of Public Health Institutional Review Board, Baltimore, Maryland; the Research & Evaluation review group of the USAID Health Care Improvement Project at University Research Co., LLC (URC), Bethesda, Maryland; and the USAID Integrated Family Health Project at URC, Bohicon, Benin.

## Results

### Demographic characteristics

Table 1 presents demographic characteristics of the study population. Antenatal consultations of 686 pregnant women were observed followed by exit interviews: 211 in the baseline intervention arm, 204 in endline intervention, 119 in baseline control, and 152 in endline control. This represented 55 providers: 26 and 29 in the intervention and control arms, respectively, although not all providers were available during both periods of data collection. On average, there were 3.9 providers per site; 49 pregnant women were observed at each site; and 12.4 women were observed for each provider. The sample size in the control arm is smaller given the lower-than-expected number of women presenting for antenatal consultation in these sites. Approximately 4% of providers working in the study's maternities were not enrolled because they were not working at the clinic during the time of study and were logistically unable to participate in study activities. Non-participation of women was observed to be rare as women were willing and interested in discussing care for themselves and their newborns. Although there were no differences in provider characteristics between study arms with respect to age, qualification, education, years working in public health, and years working at the site, group education followed by individual counseling was more common in the intervention arm in pre- and post-groups (58% and 80%) than the control arm (36% and 34%) as compared to only group or individual counseling.

**Table 1:** Characteristics of study sample

	Total	Intervention Arm			Control Arm	
		
		Pre-	Post-		Pre-	Post-
**Study Population**						

Number of sites	14	7	7		7	7
Total number of observations	686	211	204		119	152
Total number of providers	55	20	21		25	25
Counseled only at baseline	9	5	0		4	0
Counseled only at endline	10	0	6		0	4
Counseled at baseline + endline	36	15	15		21	21
Group and individual counseling (%)	55.7	58.2	79.9		35.9	33.6
Group counseling only (%)	1.3	0	4.4		0	0.7
Individual counseling only (%)	43.0	41.8	15.7		64.1	65.8

**Provider Characteristics**						

Skilled provider type (%)	86.5	83.3	95.2		92.0	87.5
Mean age (yrs)	34.6	35.3	33.6		34.4	34.2
Completed secondary education (%)	96.2	100	100		100	92
Years working in health field (yrs)	9.7	11.1	10.1		8.5	8.2
Years working at health center (yrs)	4.1	4.8	4.6		2.9	3.7

**Patient Characteristics**						

Mean age (yrs)	25.8	25.6	25.4		26.0	26.6
Mean gestational age (months)	5.9	5.9	6.0		5.9	5.8
Educational status (%, > 8 yrs)	52.9	60.0	52.0		52.9	44.1
1^st ^prenatal visit (*in current pregnancy*)	30.0	28.4	23.5		36.1	36.2
Mean no. of ANC visits (*in current pregnancy)*	2.6	2.8	2.8		2.5	2.4
Mean number of living children	1.7	1.6	1.6		1.8	1.9

The groups of pregnant women in the intervention and control arms prior to and after the intervention were similar with respect to age, months in pregnancy, number of antenatal visits, and number of living children. Women's characteristics differed by education status and first antenatal visit. Over half of the women in the baseline intervention arm (60%) had more than eight years of schooling compared to 52%, 53%, and 44% in the endline intervention, baseline control, and endline control groups, respectively. The proportion of women presenting at their first antenatal visit in the current pregnancy was higher in the control groups (36% and 36%) than in the intervention groups (28% and 24%). Mean gestational age for first-time attendees was 4.1 months across all groups (data not shown) compared to 5.9 months for the total study population. Of the observed consultations, 81% were conducted in Fon, 9% in Nagot, 2% in French, and 8% in other local languages (data not shown).

### Content of Communication

Table 2 presents changes over time in the mean percent of recommended messages provided to pregnant women by topic and study arm. On average, pregnant women in the intervention and control arm received 51% and 36%, respectively, of recommended maternal and newborn care messages during antenatal consultations at baseline. Relative improvement (Δ_I _- Δ_c_) in the mean percent of messages provided in the intervention arm was +19.6 (95% CI: 12.2, 26.9) in adjusted analyses, accounting for clustering and differences in site-, provider- and patient-level characteristics. By topic area, relative improvements in the intervention arm were observed in four out of five topics: birth and emergency preparedness (Δ_I _- Δ_c _= +17.9, 95% CI: 6.7, 29.1), danger signs during pregnancy (Δ_I _- Δ_c _= +26.0, 95% CI: 14.6, 37.4), clean delivery (Δ_I _- Δ_c _= +21.7, 95% CI: 10.9, 32.6), and for women with advanced pregnancy, newborn care (Δ_I _- Δ_c _= +26.2, 95% CI: 13.5, 38.9). In the area of general prenatal care, modest gains in communication in both study arms were observed with no significant relative improvement (Δ_I _- Δ_c _= +8.2, 95% CI: -2.6, 19.1).

**Table 2:** Changes in mean percent of messages provided during antenatal visit by topic and study arm

Mean % of messages provided	Intervention Arm			Control Arm			Difference in differences
	
	Pre-	Post-	**Differ-ence (Δ**_**i**_**)**	Pre-	Post-	**Differ-ence (Δ**_**c**_**)**	**Δ**_**i**_**-Δ**_**c **_**(95% CI)**

No. of pregnant women	211	204		119	152		
Adjusted Scores^a^							

Mean % of messages given (total)	51.4	67.9	+16.5	35.5	32.4	-3.1	+19.6 (12.2, 26.9)
Mean % of messages given (by topic^b^)							
Prenatal Care	64.5	68.6	+4.1	51.4	47.4	-4.1	+8.2 (-2.6, 19.1)
Birth preparedness	53.7	66.5	+12.9	40.5	35.4	-5.1	+17.9 (6.7, 29.1)
Danger signs during pregnancy	38.8	63.5	+24.7	24.4	23.1	-1.3	+26.0 (14.6, 37.4)
Clean delivery	49.1	70.4	+21.3	15.1	14.7	-0.4	+21.7 (10.9, 32.6)
Newborn care^c^	20.6	49.3	+28.7	3.8	6.3	+2.5	+26.2 (13.5, 38.9)
Mean % of communication techniques used	46.2	75.4	+29.2	42.7	43.0	+0.3	+28.8 (22.5, 35.2)
Mean duration of antenatal consultation^d^	18.3	23.9	+5.6	15.8	15.5	-0.3	+5.9 (3.0, 8.8)

[a] Scores adjusted for differences at baseline and correlation of observations; site- and provider-level characteristics (random effects); counseling mode; and patient age, education, first antenatal visit, and total number of antenatal visits (fixed effects). [b] Total number of messages by category include: prenatal care (n = 5), birth preparedness (n = 7), danger signs during pregnancy (n = 9); clean delivery (n = 2); newborn care (n = 6); communication techniques (n = 6). [c] Includes only women at 6 - 9 months of pregnancy. [d] Excludes additional time for women who participated in individual counseling following group session.

Table 3 presents an item analysis of key messages provided within all five topic areas. The overall proportion of women receiving communication varied from message to message. In the intervention arm, the largest improvements were observed among messages relating to identification of a skilled birth attendant, emergency planning, provision of a plastic cloth (for clean delivery) and messages relating to newborn care such as early and exclusive breastfeeding and delayed bathing. Messages with relatively high scores at baseline and only modest improvements at endline included taking antimalarials, information on nutrition for pregnant women, placing money aside, and planning for birth with a family member. No significant improvements were observed for messages concerning use of an insecticide-treated mosquito net or iron/folate supplementation during pregnancy. In the control arm, no significant changes were observed between baseline and endline.

**Table 3:** Item analysis - percent women provided with any message during antenatal visit, by topic and study arm

	Intervention Arm			Control Arm			Difference in differences	
	
	Pre-	Post-	Differ-ence (Δ_i_**)**	Pre-	Post-	Differ-ence(Δ_c_**)**	Δ_**i**_-Δ_**c**_	95% CI

No. of women (N = 686)	211	204		119	152			
Prenatal care								

Sleep under a mosquito net	76.3	74.0	-2.3	66.4	74.3	+8.0	-10.2	(-23.8, 3.4)
Take antimalarials	61.1	71.1	+9.9	53.8	55.3	+1.5	+8.4	(-6.4, 23.3)
Take iron/folic supplements	79.6	75.5	-4.1	63.0	67.8	+4.7	-8.9	(-22.5, 4.7)
Have at least 4 prenatal visits	31.8	65.7	+33.9	21.0	29.6	+8.6	+25.3	(11.2, 39.4)
Eat more and more varied	61.6	72.5	+10.9	26.1	32.2	+6.2	+4.8	(-9.5,19.0)

Birth Preparedness								

Identify place of delivery	62.6	85.3	+22.7	47.1	45.4	-1.7	+24.4	(10.3, 38.5)
Identify means of transport	56.4	86.8	+30.4	37.8	39.5	+1.7	+28.7	(14.8, 42.6)
Identified skilled attendant	32.2	71.6	+39.3	28.6	25.0	-3.6	+42.9	(28.9, 56.9)
Put money aside	73.0	84.3	+11.3	42.0	41.4	-0.6	+11.9	(-1.8, 25.6)
Plan for emergency	40.3	80.9	+40.6	28.6	30.3	+1.7	+38.9	(25.0, 52.8)
Plan with family	70.1	84.3	+14.2	47.1	48.7	+1.6	+12.5	(-1.4, 26.4)
Identify a blood donor	50.7	68.6	+17.9	30.3	28.3	-2.0	+19.9	(5.3, 34.4)

Danger signs during pregnancy								

Vaginal bleeding	51.2	71.1	+19.9	27.7	32.9	+5.2	+14.7	(0.2, 29.3)
Convulsions	15.2	53.9	+38.7	4.2	7.9	+3.7	+35.0	(23.6, 46.5)
Fever	53.1	71.6	+18.5	32.7	32.2	-0.5	+19.0	(4.4, 33.6)
Water loss	49.8	71.6	+21.8	21.8	28.3	+6.4	+15.4	(1.1, 29.6)
Abdominal pains	50.2	72.5	+22.3	28.6	28.3	-0.3	+22.6	(8.2, 37.0)
Severe headaches	36.0	68.1	+32.1	21.8	25.0	+3.2	+29.0	(14.9, 43.0)
Blurred vision	23.2	66.1	+43.0	15.1	14.5	-0.6	+43.6	(30.8, 56.4)
Swelling of limbs	35.1	57.4	+22.3	14.3	13.8	-0.5	+22.8	(9.3, 36.2)
Diminished fetal movement	29.9	57.4	+27.5	10.1	18.4	+8.3	+19.2	(5.8, 32.5)

Clean Delivery								

Bring plastic cloth	27.5	66.7	+39.2	0.8	2.6	+1.8	+37.4	(26.1, 48.7)
Bring 5 clean towels	65.9	81.9	+16.0	18.5	21.1	+2.6	+13.4	(0.4, 26.4)

Immediate newborn care^a^								

Skin-to-skin contact	21.5	46.7	+25.1	4.4	5.7	+1.3	+23.8	(8.2, 39.3)
Initiation of immediate BF	20.8	58.3	+37.6	5.9	11.5	+5.6	+32.0	(15.9, 48.0)
Avoid prelacteal foods/exclusive BF	9.2	55.0	+45.8	5.9	10.3	+4.4	+41.3	(26.7, 55.9)
Delayed bathing	3.1	41.7	+38.6	2.9	2.3	+0.6	+39.2	(27.0, 51.5)
Clean cord care	4.6	37.5	+32.9	1.5	5.7	+4.2	+28.6	(15.9, 41.3)
Thermal protection	19.2	48.3	+29.1	2.9	8.0	+5.1	+24.0	(8.6, 39.4)

Communication Technique								

Presents the subject	72.5	98.5	+26.0	73.1	81.6	+8.5	+17.5	(6.3, 28.8)
Poses questions to determine current knowledge of pregnant woman	61.1	99.0	+37.9	54.6	59.9	+5.2	+32.6	(19.8, 45.5)
Uses cards or other visual aids	28.4	99.5	+71.1	16.8	19.7	+2.9	+68.1	(57.3, 79.0)
Verifies understanding	55.0	98.5	+43.6	52.9	61.8	+8.9	+34.7	(21.7, 47.6)
Motivates to adapt behaviors	69.2	96.1	+26.9	26.9	38.2	+11.2	+15.6	(3.1, 28.1)
Asks woman if she has questions	72.0	97.1	+25.0	50.4	59.2	+8.8	+16.2	(3.6, 28.9)

[a] Includes only women at 6 - 9 months of pregnancy.

### Communication Techniques and Duration

Overall mean performance in communication techniques of health providers was 46% and 43% at baseline in intervention and control arms, respectively (Table 2). The relative mean percent of communication techniques used was significant in the intervention arm (Δ_I _- Δ_c _= +28.8, 95% CI: 22.5, 35.2). At the item level, providers presented the subject to be discussed in the majority of consultations, and baseline measures of techniques such as posing questions to ascertain current knowledge, verification of understanding, and asking the woman if she had questions were observed at moderate levels in intervention and control arms. These techniques (as well as others that were used less frequently) remained unchanged in the control arm, but increased significantly in the intervention arm. The two most notable increases in techniques used were use of a visual aid - the intervention's counseling job aids - and verification of understanding, which included a summary of key messages following the consultation.

These improvements were associated with increases in consultation duration. Each session lasted an average 18 minutes at baseline in both study arms and significantly increased to 24 minutes in the intervention arm (Δ_I _- Δ_c _= +5.9, 95% CI: 3.0, 8.8). The observed additional time appears to have been associated with increased communication, although time spent in clinical examination versus communication was not measured systematically.

### Maternal Knowledge

Improvements in knowledge among pregnant women were observed in the area of birth preparedness, recognition of danger signs, and clean delivery after controlling for differences at baseline, correlation of data, and level-specific characteristics (Table 4). At baseline, 21% and 26% of pregnant women in the intervention and control arm, respectively, could correctly identify at least three components of birth and emergency preparedness with a relative improvement in the intervention arm of +23.6 (95% CI: 9.8, 37.4). There was also a significant relative improvement in the proportion of women who could identify at least three danger signs during pregnancy that needed the urgent attention of a health professional (Δ_I _- Δ_c _= +28.7, 95% CI: 14.2, 43.2). Signs such as abdominal pains (92%), fever (88%), and heavy bleeding (79%) were most commonly reported by women in the intervention arm (not shown). A relatively lower proportion of women indicated water loss (68%), blurred vision (34%), or absent or diminished fetal movement (24%) as a danger sign. Convulsions (21%) and swelling of the limbs (13%) were least commonly considered indicative of danger (data not shown). Significant relative improvements were also observed for correct knowledge of both clean delivery items (Δ_I _- Δ_c _= +31.1, 95% CI: 19.4, 42.9). No significant relative improvement was observed in maternal knowledge regarding general prenatal care (Δ_I _- Δ_c _= -6.4, 95% CI: -21.3, 8.5) or newborn care (Δ_I _- Δ_c _= +12.7, 95% CI: -6.1, 31.5). As compared to the control arm, the overall mean number of correct responses across all topic areas improved significantly in the intervention arm (Δ_I _- Δ_c_= +2.9, 95% CI: 1.9, 3.9).

**Table 4:** Changes in maternal knowledge by topic and study arm

Percentage (%) of women with correct responses	Intervention Arm			Control Arm			Difference in differences

	
	Pre-	Post-	(Δ_i_)	Pre-	Post-	(Δ_c_)	Δ_i _- Δ_c _(95% CI)
No. pregnant women	211	204		119	152		
Adjusted Scores^a^							

≥ 3 messages in prenatal care	49.8	62.7	+12.9	26.5	45.8	+19.3	-6.4 (-21.3, 8.5)
≥ 3 messages in birth preparedness	20.5	47.9	+27.4	25.7	29.5	+3.8	+23.6 (9.8, 37.4)
≥ 3 danger signs during pregnancy	55.3	88.8	+33.2	40.4	44.9	+4.5	+28.7 (14.2, 43.2)
= 2 messages in clean delivery	15.7	49.2	+33.5	2.5	4.9	+2.4	+31.1 (19.4, 42.9)
≥ 3 messages in newborn care^b^	32.5	57.7	+25.2	21.4	33.9	+12.4	+12.7 (-6.1, 31.5)

Mean # correct responses	8.6	12.6	+3.9	7.7	8.7	+1.0	+2.9 (1.9, 3.9)

[a] Scores adjusted for differences at baseline and correlation of observations; site- and provider-level characteristics (random effects); counseling mode; and patient age, education, first antenatal visit, and total number of antenatal visits (fixed effects). [b] Includes only women at 6 - 9 months of pregnancy.

### Provider Perceptions

Table 5 lists provider perceptions regarding use of the counseling job aids within three topic areas: perceived advantages, perceived disadvantages, and suggestions to improve overall use and effectiveness. The three most commonly reported advantages to using the aids were that they helped women retain information given the images of key signs or practices; they helped the provider remember what topics to discuss during the antenatal session; and the perceived time required for explaining a practice was less since the images well depicted the desired communication goal. Providers using the counseling job aids also noted that having them allowed workers to improve their skills over time and that women presenting at the clinics also appreciated and showed interest in the counseling cards.

**Table 5:** Provider perceptions regarding use of counseling job aids (Intervention arm only)

	**Advantages to using counseling job aids**:		**Disadvantages to using counseling job aids**:		**Suggestions to improve use of counseling job aids**:

-	Pregnant women better retain messages*	-	Requires additional time which delays when the women leave the health center	-	Improve durability of the cards
-	Health workers review all communication elements	-	Requires additional time for providers*	-	Extend duration of training in use of the cards
-	Increases speed in which women grasp key messages	-	Too many cards; difficult to organize	-	Decrease number of cards used in each module*
-	Cards function as a reminder ("job aid') for the health worker of key messages*			-	Increase number of messages per card
-	Relieves provider of burden to explain messages without images				
-	Less time is needed for explaining because the cards' images assist in comprehension among women (saves time)*				
-	Allows provider to master material over time				
-	Depicts danger signs and consequences of poor practices that women can visualize				
-	Women appreciate the cards				

Note: The symbol (*) denotes that providers commonly gave the response. All providers responded "yes" when asked whether they thought the counseling job aids should be introduced at other sites.

On the other hand, providers remarked that the required additional time to use all of the counseling cards in a given module, including verification of prior knowledge in preceding modules, was a disadvantage that delayed women's departure from the antenatal clinic. Some providers suggested that the number of counseling cards be decreased in tandem with an increase in the number of messages per card while improving their durability as well from the original laminated format. A few workers proposed that the training session be extended from its current three-day module to provide more time for discussion and practice. All providers recommended that the counseling job aids be introduced at other sites to strengthen antenatal education.

## Discussion

Health information-sharing is an essential element of focused antenatal care. Yet, baseline study findings showed that although women had multiple antenatal visits, they were not profiting entirely from effective communication for care during and after pregnancy. While later pregnancy stage was correlated with increased knowledge at baseline, overall knowledge was poor despite repeated clinical contacts. Seventy percent of women in the study had more than one prior antenatal visit in the current pregnancy, and the majority had previous pregnancies. Yet, fewer than a quarter of women correctly identified components of birth preparedness and danger sign recognition compared to over a half to two-thirds at endline. Such low levels of awareness indicated a missed opportunity for health promotion during pregnancy.

Results of this study showed that introduction of a job aids-focused intervention can improve quality of counseling and, in turn, maternal understanding when job aids are combined with training, field support, and site-level organizational changes. Findings demonstrated that communication content and techniques significantly improved following the intervention in such areas as birth preparedness, danger sign recognition, clean delivery, and newborn care. Improved communication was also associated with improved maternal knowledge in birth preparedness, danger signs, and delivery care. Maternal knowledge of newborn care also increased in the intervention arm, although this was not significant after adjusting for changes in the control arm. Few studies have examined what specific information is provided antenatally to women within the context of maternal and newborn care in a resource-poor African setting. This implementation research enabled intermediate results to be linked with communication processes and provided information on the relative effectiveness of a set of performance support tools for health care personnel. The results of the study support findings of earlier studies in which clinic-based health education coupled with job aids effectively improved quality of communication [24] and patient comprehension [31].

Although counseling quality improved in most topic areas, no relative gains in counseling were observed for messages regarding general prenatal care. This may have resulted because baseline levels in provider counseling as well as maternal knowledge were highest in this topic area. Providers in the intervention arm may not have placed as much priority on improving communication given perceptions that maternal knowledge was already adequate. Nonetheless, the job-aid focused intervention's observed improvements across most topics in the span of one clinical visit is encouraging and suggests that, following job aid-supported counseling, pregnant women are more equipped to make informed decisions regarding their health and that of their newborn.

Several factors likely contributed to the observed improvement in addition to provider training and field support. One potential factor is that the maternal and newborn care counseling aids were adapted to the Beninese environment and were easy to use. In their design as a practical aid for communication, they were consistent with existing skills and practices, easy to "try out," organized in modules (to prioritize messaging), and yielded visible results. These features have been shown to enhance the implementation of new practices [32,33]. The tools also provided clarity to provider communication goals and used locally relevant graphics to enhance the engagement of low-literate women [34].

A second factor relates to implementation of the intervention. Uptake in the use of job aids was high among providers, and the organizational sessions included the head nurse-midwife regardless of the extent of her involvement in antenatal communication. This was done to facilitate the introduction of site-level organizational changes needed to improve communication, particularly for organizing counseling sessions and ensuring adequate feedback mechanisms. Evidence shows that local leadership enhances the implementation of new practices [35], which is consistent with the programmatic experience of this study.

Despite these achievements, the job-aid focused intervention was unable to mitigate all implementation barriers, which possibly explains why gains were moderate in some areas. During interviews, providers reported that lack of or limited dedicated space for counseling remained a challenge along with language barriers (in areas with multiple dialects). In addition, clinical tasks, such as managing deliveries, disrupted or prevented high quality antenatal communication. Lack of time was the most commonly reported barrier as providers recognized that good communication takes time. Recent studies have shown that duration of antenatal consultations is low in many developing countries with even less time spent on communication [6,11]. Within the intervention arm, there was a significant increase in the duration of antenatal consultations following introduction of the counseling job aids. Such an increase may not always be problematic [18]. In fact, the relatively modest increase was not perceived to be unmanageable, but the cited time barrier by providers for effective communication does deserve consideration. It is interesting to note that some health workers indicated that the images actually reduced the time needed for explanation, allowing them to increase the number of messages. However, the challenge of communicating effectively in relatively short periods of time still needs to be addressed.

Particularly in facilities where health personnel are understaffed or have multiple clinical responsibilities, one alternative may be to explore the feasibility and effectiveness of expanding the role of less skilled health workers who may have fewer time constraints. Nearly three-fourths of women in the intervention arm said they would have preferred having the provider spend more time with them for counseling (data not shown). This was higher than the proportion of women at baseline, perhaps resulting from an increased provision of information which raised interests and discussion preferences. It is also important to note that while the job aids-focused intervention was effective in standardizing communication, one drawback may be over-reliance on the tools that results in overly structured, less-individualized sessions. Efforts are needed to ensure that communication is still patient-centered and that counseling job aids are used as an aid, not a verbatim guide for communication.

### Limitations

These findings should be interpreted in light of the study's limitations. One, due to the nature of the intervention, it was not operationally feasible to blind the data collection team regarding the intervention or control status of participating sites. Although supervisors observed a sample of counseling sessions for quality control purposes, some reporting biases may have occurred. Health workers may likewise have altered their behavior in response to being observed, which may have inflated documented performance levels. It is possible that cross contamination also led to an under-estimation of the relative effect, although this is unlikely given efforts to only implement job aids-focused activities in intervention sites.

Two, the study did not assess whether improved counseling and maternal knowledge, in turn, led to improved home care practices among women, which was the basis for much of the information shared. Studies have shown that increased knowledge is not necessarily linked with behavior change [36], and there is still limited empirical demonstration of the effectiveness of some communication goals, such as birth preparedness, which was included in this study [37]. However, the research does demonstrate the intervention's direct effects on health worker behavior and maternal knowledge, which provides important insight on programmatic strategies to improve health outcomes. The counseling job aids were valued in their design as a practical tool in the intermediate step of communication, which arguably is crucial in empowering individual women to safeguard their health and that of their newborn.

Other possible limitations are that despite randomization of sites and similarities in demographic characteristics, there were significant differences in baseline quality of counseling between study arms, which may have accounted for differences in outcomes at endline. To adjust for this, data were evaluated using a difference-in-differences analysis, although it is possible that the observed intervention effect would be lower (or higher) in sites with dissimilar baseline performance levels. No specific reasons were identified to explain differential performance levels at baseline between the two study arms. In addition, several study sites were participating in a quality improvement initiative at the time of the study. As a result, they may have been more willing to introduce an improvement strategy and have been more apt to benefit from introduction of such as compared to sites without experience in quality improvement. Sub-analyses found no differences in counseling performance between sites participating in an improvement collaborative versus those who were not. This may have resulted since the improvement collaborative had not focused on antenatal counseling as part of its scope. Participation in the collaborative did not vary significantly between intervention and control arms. By design, the study was likewise unable to differentiate between the unique effect of the job aids in the intervention group as compared to the job aid training, organizational changes, and field support. Our findings thus represent an evaluation of a job aids-focused package of interventions than the tools alone. Lastly, the study did not assess the extent to which improved quality of counseling and maternal knowledge were maintained over time or enhanced as a result of repeated use of the counseling job aids throughout a woman's pregnancy. Qualitative research such as in-depth interviews with women participants would provide greater insight on effectiveness of communication over time.

### Implications

Several policy and programmatic implications emerge from the study's findings. One, this study demonstrates that job aids with training, field support, and organizational change are an effective strategy for improving provider communication and should be integrated into routine antenatal care strategies. However, widespread implementation of the counseling aids will need to factor in how changes in communication activities influence other service areas. Our experience revealed that improved communication led to longer antenatal consultations. While it is plausible that increased efficiency using the cards may save time in the long run, such benefits have not yet been shown. Furthermore, broad scale implementation of the cards will require a functional supply management system beyond mechanisms used for this study, as well as consideration of the social norms that influence provider-patient interactions.

A second implication for practice relates to quality improvement. This study found that improving quality of communication was associated with increased maternal understanding in a relatively short period and that building capacity of health personnel with appropriate performance support was crucial in the process. Findings suggest that antenatal care programs should target strengthening communication to pregnant women as part of systematic improvement strategies. This should be combined with on-going quality assessment that identifies the kind of information given to women and its impact on their behavior and understanding.

Lastly, findings highlight that in practice women in advanced pregnancy can and need to be targeted for advice on newborn care. International guidelines emphasize the need to promote newborn care during the antenatal period, not just after birth [38]. Recent evidence demonstrates that antenatal health communication to promote evidence-based newborn care practices is effective in averting newborn mortality in developing countries [39]. The magnitude of antenatal clinical coverage in the developing countries means that pregnant women in many contexts can be advised on care of the newborn prior to birth along with maternal care during pregnancy and birth preparation.

## Conclusions

Antenatal health counseling is an important strategy to promote awareness of maternal and newborn health during pregnancy, yet quality of communication is often poor and understudied. Findings from this study indicate that use of a job aids-focused intervention can be an effective strategy to improve quality of antenatal communication as well as maternal understanding. Efforts are needed to address time constraints and other barriers to communication, including introduction of on-going quality assessment and appropriate mechanisms for scale-up.

## Competing interests

The authors declare that they have no competing interests.

## Authors' contributions

LJ conceived and designed the study, developed the data collection instruments, supervised data collection, performed the statistical analysis, and wrote all versions of the manuscript. ASY and JA participated in the testing and finalization of the data collection instruments, carried out the job aids training, participated in data collection, coordinated field implementation, reviewed the study results, and made contributions to the manuscript. MA participated in the design of the job aids and associated training, reviewed study results, and made contributions to the manuscript. All authors read and approved the final manuscript.

## Pre-publication history

The pre-publication history for this paper can be accessed here:

http://www.biomedcentral.com/1471-2393/10/75/prepub
